# The Emerging Role of Interleukin-(IL)-11/IL-11R in Bone Metabolism and Homeostasis: From Cytokine to Osteokine

**DOI:** 10.14336/AD.2023.0306

**Published:** 2023-12-01

**Authors:** Bingzi Dong, Jingjing Zhu, Xian Chen, Hongyuan Jiang, Yujie Deng, Lili Xu, Yangang Wang, Shufa Li

**Affiliations:** ^1^Department of Endocrinology and Metabolism, The Affiliated Hospital of Qingdao University, Qingdao, China; ^2^Department of Clinical Laboratory, The Affiliated Hospital of Qingdao University, Qingdao, China; ^3^Department of Sports Medicine, The Affiliated Hospital of Qingdao University, Qingdao, China

**Keywords:** Interleukin (IL)-11, bone formation, osteoblast differentiation, osteoclast, bone-derived hormones, adipogenesis

## Abstract

Interleukin-(IL)-11 is a cytokine involved in hematopoiesis, cancer metastasis, and inflammation. IL-11 belongs to the IL-6 cytokine family, binding to the complex of receptors glycoprotein gp130 and the ligand-specific-receptor subunits (IL-11Rα or their soluble counterpart sIL-11R). IL-11/IL-11R signaling enhances osteoblast differentiation and bone formation and mitigates osteoclast-induced bone resorption and cancer bone metastasis. Recent studies have shown that systemic and osteoblast/osteocyte-specific IL-11 deficiency leads to reduced bone mass and formation, but also adiposity, glucose intolerance, and insulin resistance. In humans, mutations of IL-11 and the receptor IL-11RA genes are associated with height reduction, osteoarthritis, and craniosynostosis. In this review, we describe the emerging role of IL-11/IL-11R signaling in bone metabolism by targeting osteoblasts, osteoclasts, osteocytes, and bone mineralization. Furthermore, IL-11 promotes osteogenesis and suppresses adipogenesis, thereby influencing the fate of osteoblast/adipocyte differentiation derived from pluripotent mesenchymal stem cells. We have newly identified IL-11 as a bone-derived cytokine that regulates bone metabolism and the link between bone and other organs. Thus, IL-11 is vital in bone homeostasis and could be considered a potential therapeutic strategy.

## Introduction

Interleukin-11 (IL-11) is a member of the cytokine interleukin IL-6 family. It binds to the receptor complex of a ligand-specific receptor subunit (IL-11Rα or its soluble counterpart sIL-11R) and the transmembrane glycoprotein β-subunit gp130. It shares gp130 with the other cytokines from the IL-6 family (IL-6, leukemia inhibitor factor (LIF), oncostatin M (OSM), and ciliary neurotrophic factor (CNTF)) [1].

IL-11 is involved in hematopoiesis. In synergy with other cytokines, it promotes bone marrow hematopoiesis, megakaryocyte maturation, and platelet formation [2]. It is currently used as a platelet-stimulating factor in clinical practice. Recombinant IL-11 (Oprelvekin) has been approved by the US Food and Drug Administration for the treatment of thrombocytopenia in humans [3]. IL-11 has a potential therapeutic effect on inflammatory bowel disease [4] and rheumatoid arthritis based on its anti-inflammatory effects as revealed by small-scale clinical and animal studies [5, 6]. Recently, the role of IL-11 in various physiological and pathological processes has been investigated.

In this review, we aimed at updating and summarizing the role of IL-11 in bone metabolism and homeostasis, including bone development, formation and resorption, and cancer bone metastasis. We identified IL-11 as a novel bone-derived cytokine that affects bone remodeling and the link between bone and other organs. It enhances osteoblastogenesis and suppresses adipogenesis. Thus, this insight provides a novel perspective on the therapeutic potentials of IL-11.

## Structure of IL-11/IL-11R

IL-11 is secreted by several mesenchymal-origin cells, including osteoblasts, osteoclasts, chondrocytes, fibroblasts, leukocytes, epithelial cells, keratinocytes, and synoviocytes [7]. The protein is encoded by the IL-11 gene, which contains five coding exons and four introns and is located on chromosome 19q13. The promoter region of IL-11 contains binding sites for several transcriptional factors, including activator protein-1 (AP-1), Runt-related transcription factor 2 (Runx2), and Smad [8]. The transcription and expression of IL-11 are mainly regulated by extracellular signal-regulated kinases (ERKs) and p38 mitogen-activated protein kinase (MAPK) signaling pathways via the AP-1 family of transcriptional factors [9, 10].

IL-11 binds to IL-11Rα and sIL-11R, thereby influencing the physiological and pathological processes. The formation of IL-11/sIL-11R complexes two molecules of gp130 dimerization, leading to the activation of the Janus kinase/signal transducer and activator of transcription (JAK/STAT) and MAPK signaling cascades. This procress is characterized as “*trans*-signaling” [11, 12]. ADAM10 is a metalloprotease that releases the IL-11R ectodomain from the cells. The serine proteases neutrophil elastase (NE) and autoantigen proteinase 3 (PR3) also cleave IL-11R, in combination with IL-11, and induce *trans*-signaling downstream [13]. In addition, IL-11 trans-signaling is actived via Rhomboid-Like 2 (RHBDL2)-derived sIL-11R [11]. Soluble forms of both IL-6 receptor (IL-6R) and IL-11Rα have been identified. Signaling can be initiated through membrane-bound IL-6R (classic signaling) and soluble forms of IL-6R (trans-signaling) [14, 15]. Transmembrane gp130 is involved in some cytokine-mediated cellular responses and acts as a signal-transducing receptor subunit. IL-11 “*trans*-presentation” signaling has been identified to occur through IL-11R binding to gp130. IL-11 binds to cleaved sIL-11R and induces cellular proliferation via gp130 in a STAT3-dependent manner. IL-11: sIL-11R takes the overlapping binding site of the common signal transduction gp130 to activate STAT3 and STAT1 via the JAK/STAT pathway [16,17]. Studies *in vitro* demonstrate that IL-11 promotes gastric tumorigenesis and affects fertility through classic signaling pathways [18, 19]. Therefore, the role of IL-11 trans signal transduction *in vivo* requires further investigation.

## Downstream signaling of IL-11/IL-11R

IL-11 regulates cellular function mainly through three downstream signaling pathways: the JAK/STAT3, Ras/Raf/MAPK and phosphatidyl-inositol 3 kinase (PI3K/Akt) pathways [20-22].

JAK/STAT signaling is the most well-known downstream pathway of IL-11. IL-11 signal transduction that involves gp130 mediated by the activation of STAT3 and a relatively low level of STAT1 [1, 23]. After IL-11 binds to its receptors, JAKs form a complex with the adapter protein. STATs then become tyrosine-phosphorylated, leading to their dimerization and dissociation from the receptor complex. Activated JAK kinase may lead to tyrosine phosphorylation and stimulation of the STAT family transcriptional factors [22]. Tyrosine phosphorylation of STAT3 and STAT1 are induced by IL-11, though the latter occurs at higher concentrations in a dose-dependent manner [24]. In multiple sclerosis, the activation of STAT3 by IL-11 influences oligodendrocytes, whereas the activation of STAT1 predominates in DCs, leading to apoptosis and damping of the inflammatory response. STAT is phosphorylated by JAK on specific tyrosine residues, and STAT homo- and heterodimers dock via the SH2 domain [25]. Phosphorylated STATs dimerize, translocate into the nucleus, and trigger the transcriptional modulation of target genes [26].

IL-11 and its receptor IL-11R are expressed in fibroblasts. They drive fibrogenic protein synthesis via non-canonical ERK-dependent autocrine signaling [27, 28]. Administering a high concentration of rhIL-11 promotes the formation of the active GTP-bound form Ras and coupled with the growth factor receptor binding protein 2 (Grb2)/son of sevenless complex, thereby initiating the Ras signaling pathway in adipocytes [29]. This pathway promotes the activation of Raf kinases, which, in turn, phosphorylate MEK kinases resulting in MAPK activation and subsequent regulation of intracellular targets [30]. Thus, IL-11 is transduced in part through the Ras/Raf/MAPK signaling pathway [30, 31].

**Figure 1.**
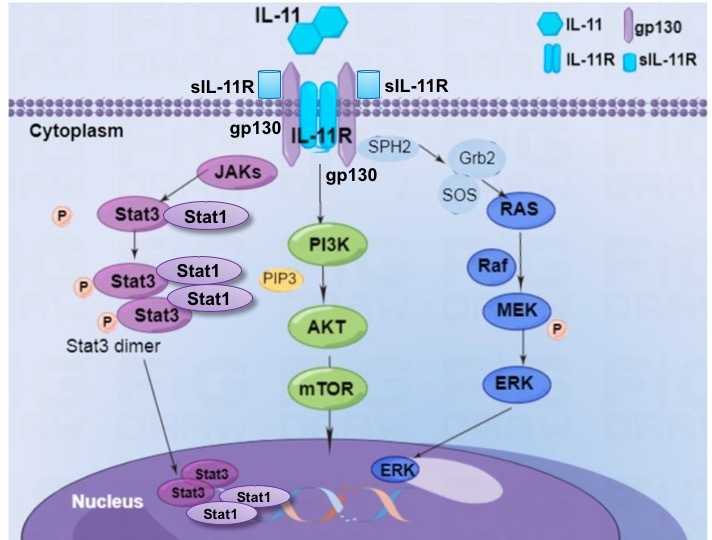
**The signaling pathway downstream of IL-11/IL-11R**. In classic signaling, IL-11 binds to the unique receptor IL-11Rα and signal-transducing receptor gp130. The IL-11/IL-11R complex recruits a homodimer of the signal-transducing receptor gp130, resulting in activation of downstream signaling. In trans-signaling, the gp130 dimer is induced by IL-11 bound to the soluble IL-11R (sIL-11R). After IL-11 binds to receptors, JAKs forms a complex with the adapter protein. The STATs then become tyrosine phosphorylated and translocates into the nucleus and trigger the transcriptional modulation of target genes. IL-11 drives non-canonical ERK-dependent autocrine signaling for fibrogenic protein synthesis, then promotes the formation of the active Grb2/SOS complex, then phosphorylate MEK kinases, and results in MAPK activation. IL-11 can also activate the PI3K/AKT pathway, independent of the tyrosine-phosphorylation of gp130, in tumor metastasis. The downstream signaling cascades are activated, including JAK/STAT1/STAT3, PI3K/Akt, and Ras/Raf/MAPK signaling in physiological and pathological condition, to promote target gene transcription.

IL-11 also activates the PI3K/AKT pathway independent of the tyrosine phosphorylation of gp130. This mechanism is shared by IL-11 and IL-6. PI3K inhibitor and siRNA-STAT3 mitigate the expression of matrix metalloproteinase (MMP)-13 induced by rhIL-11, revealing the involvement of PI3K/AKT and JAK/STAT3 signal transduction pathways [32]. IL-11 increases cell proliferation via MEK- and PI3K-dependent signaling pathways, but attenuates cell apoptosis only through the PI3K-dependent signaling pathway [33]. The expression of p-Akt and the apoptosis-related proteins Bcl-2 (anti-apoptotic protein), Bcl-xl (anti-apoptotic protein), and Bax (pro-apoptotic protein) are downregulated in IL-11 knockdown cells, revealing that IL-11 activates the PI3K/Akt signaling pathway in the treatment of radiotherapy-resistant cervical cancer [32, 34]. IL-11 upregulates MMP-13 expression by activating PI3K, Akt, and AP-1 signaling pathways that subsequently enhances MMP-13-induced tumor metastasis [33]. Thus, IL-11 is involved in tumorigenesis via the PI3K/Akt pathway (Fig. 1). Furthermore, the PI3K/Akt pathway is activated by IL-11, particularly in cancer metastasis and tumorigenesis.

## The emerging role of IL-11 in bone homeostasis

IL-11 plays a vital role in bone development and remodeling [35]. Bone remodeling is essential for homeostasis and renewal and occurs in cortical and trabecular bones. It consists of two phases: bone formation and bone resorption. Osteoclasts lead to resorption of damaged bone, and osteoblasts induce bone formation. forming the multicellular unit termed as “bone remodeling unit”. The initial remodeling signal recruits osteoclast precursors to the remodeling site. In the following reversal phase, osteoclasts disappear, and osteoblasts replace osteoclasts in the formation phase. During the remodeling cycle, the resting bone surface is maintained until the next wave of remodeling. This complex interaction between osteoblast-associated bone formation and osteoclast-induced bone resorption is important for maintaining bone remodeling and homeostasis. The balance between bone formation and resorption is crucial for sustaining bone mass and homeostasis [36]. Bone remodeling is regulated by local and systemic factors, including estrogen, calcitonin, parathyroid hormone and 1,25(OH)_2_-vitamin D_3_, those hormones involving in bone resorption and formation [37]. In a recent study, IL-11 regulates all types of cells involved in bone homeostasis, including osteoblast, osteoclast, and osteocyte.

## IL-11 in osteoblast, osteocyte, bone formation and bone mineralization

IL-11/IL-11R is involved in osteoblast-mediated osteogenesis, thereby influencing bone and skeletal structure and shape. IL-11R knockout (IL-11R^-/-^) mice exhibited a decline in osteoblasts count and bone formation, but showed an increase in trabecular bone volume [38]. In contrast, *ex vivo* differentiation of osteoblast precursors derived from the bone marrow of IL-11R^-/-^ mice revealed similar osteoblast maturation or osteoblast-mediated mineralization. This indicates the involvement of other cells within the bone microenvironment [38]. Similar to the other subunits of the IL-11 receptor, gp130 knockout (Gp130^-/-^) mice also showed reduced osteoblast count and bone formation. However, they exhibited decreased trabecular bone volume [39]. Gp130^-/-^ mice died perinatally and exhibited delayed bone development and short skeleton in the embryo [39]. Interestingly, a conditional knockout of gp130 in osteoblasts and osteocytes also resulted in decreased bone formation and reduced trabecular bone mass, despite a normal number of osteoblasts [40]. This reveals that bone formation and remodeling require IL-11 signaling through the Gp130/IL-11Rα receptor complex.

**Table 1:** The phenotypes of mutant IL-11 ligand and receptors in animal models and human diseases.

		Bone mass	Bone formation	Bone resorption	Adiposity	Human diseases
**Ligand IL-11**						
**IL-11 deletion**	IL-11-/- global [41]	Bone mass↓	Bone formation↓ Osteoblast cell number↓ Osteoblast differentiation ↓	Bone resorption→ Osteoclast Number→	Systemic AT↑ Bone marrow adiposity↑ Impaired glucose tolerance and insulin resistance	Height↓ [96-98]
	Osteoblast specific IL-11-/- (Ocn-Cre) [41]	Bone mass↓	Bone formation↓ Osteoblastogenesis↓	Bone resorption→ Osteoclast Number→	Systemic AT↑ Impaired glucose tolerance and insulin resistance	--
	Adipocyte specific IL-11-/- (Adipo-Cre) [41]	Trabecular and cortical BMD→	Bone formation→	Bone resorption→	Adipose tissue→ Normal glucose metabolism	--
**IL-11 overexpression**	IL-11 Transgenic [42]	Bone mass↑ against aging, cortical thickness↑	Bone formation↑ Osteoblast number↑ Osteoblastogenesis↑ and bone mineralization↑ *ex vivo*	Bone resorption→ Osteoclast Number→	Adipogenesis↓ from BMSC *ex vivo*	--
**Receptor**						
**IL-11Receptor**	IL-11R null [7,35,38]	Trabecular bone mass↑ Bone length↓	Bone formation↓ Osteoblast number↓ Osteoblast differentiation→ *in vitro*	Bone resorption↓ osteoclastogenesis↓ osteoclast number↓ maturation↓	Bone marrow adiposity↓	Craniosynostosis, dental abnormalities, and digit malformations [100-102]
**Gp130**	Gp130 null, gp130-STAT3 deletion [39]	Trabecular bone mass↓	Bone formation↓ Osteoblast number↓	Large osteoclast		--
	osteoblast/osteocyte specific -/- (Osx-Cre, Dmp1-Cre) [40]	Trabecular bone mass↓	Bone formation↓ Ob number→	Bone resorption→ Osteoclast Number→		--
	Osteoclast specific -/- (Ctsk-Cre) [69]	Trabecular bone mass↓ Cortical growth↓	Bone formation↓	Bone resorption→ Osteoclast number→		--

Recently, Dong et al. demonstrated that systemic IL-11 knockout (IL-11^-/-^) result in reduced vertebral and femoral bone mineral density (BMD), bone formation, osteoblast count, and expression of osteoblastogenic genes. However, the osteoclast count, serum bone resorption markers, and expression of osteoclastogenic genes were comparable to those in WT mice. Thus, the reduced bone mass in IL-11^-/-^ mice results from decreased bone formation, but not bone resorption. In addition, IL-11^-/-^ mice showed suppressed bone formation in response to mechanical loading due to the enhanced expression of Wnt inhibitors and suppression of Wnt signaling. Furthermore, general adiposity and bone marrow adipose tissue were increased in IL-11^-/-^ mice [41]. Osteoblast/osteocyte-specific IL-11 deletion in osteocalcin-Cre; IL-11fl/fl mice resulted in reduced serum IL-11 levels, blunted bone formation under mechanical loading, and increased systemic adiposity, similar to systemic IL-11^-/-^ mice. Moreover, systemic and osteoblast/osteocyte-specific IL-11 deficiencies led to the reduced bone mass and bone formation in response to mechanical loading, as well as increased adiposity, glucose intolerance, and insulin resistance [41]. Therefore, bone-derived IL-11 acts as an osteokine, participating in systemic regulation of bone metabolism and other organs. On contrast, IL-11 overexpressed-transgenic (Tg) mice exhibited enhanced bone formation, increased cortical thickness and bone strength, indicating that IL-11 is involved in endochondral bone formation [21, 42]. IL-11 Tg mice showed greater bone mass preservation and resistance to age-related bone loss [42] (Table 1). IL-11^-/-^ mice showed reduced bone mass, osteoblast count, and bone formation, similar to the phenotypes of gp130^-/-^mice, but contrary to the phenotypes of IL-11 Tg mice. However, IL-11R^-/-^ mice showed increased bone mass. These differences as well as the mechanisms underlying them are unclear. This could be because IL-11R null compensates the other subunit gp130 function, or it alters its competition for the shared subunit. However, further investigation is required to ascertain this hypothesis.

**Figure 2.**
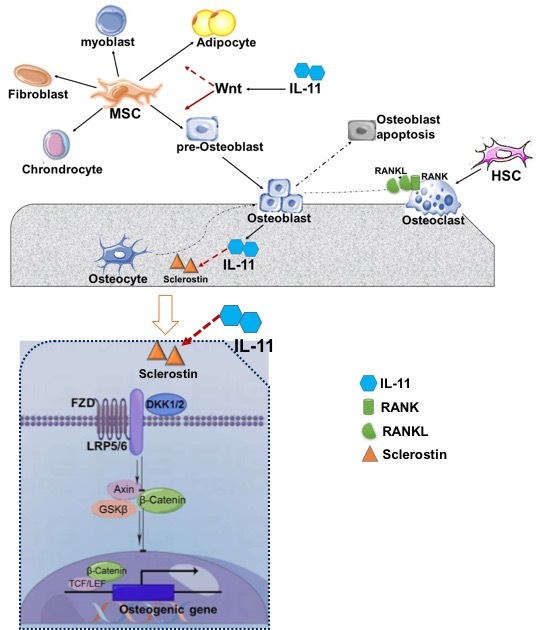
**The effect of IL-11 on shifting cell fates from mesenchymal stem cells**. Mesenchymal stem cells can be differentiated into osteoblast, adipocyte, fibroblast, chondrocyte, myoblast and so on. IL-11 promotes osteoblast differentiation and osteogenesis, while suppresses adipocyte differentiation and adipogenesis via activation of Wnt/β-catenin signaling pathway. IL-11 suppresses the Wnt inhibitors sclerostin and Dkks. IL-11 regulates the osteoblast/adipocyte differentiational cell-fate from mesenchymal stromal cells.

Previous studies have also demonstrated that IL-11 is an important regulator of mechanical stress-induced osteoblast differentiation through canonical Wnt/β-catenin signaling [43]. Wnt/β-catenin signaling promotes osteoblast differentiation and proliferation, maintains bone marrow stromal cell (BMSC) self-renewal, and mediating the crosstalk between chondrocytes and osteoblasts in bone growth plates [44, 45]. Wnt inhibitors, including sclerostin (SOST) and Dickkopf (DKK) 1/2, bind to the Wnt protein or prevent the interaction between Wnt protein and its co-receptors, thereby preventing β-catenin translocation and inhibiting osteogenic differentiation and bone formation [46]. IL-11 promotes bone formation in response to mechanical stress in part. The expression of IL-11 in bone tissues is upregulated by mechanical loading [35]. Mechanical stress rapidly enhances FosB transcription into deltaFosB, then forms a heterodimer with JunD and binds to the IL-11 gene promoter, enhancing *IL-11* gene transcription and expression [47, 48]. Mechanical stress also activates the Smad-1 pathway through protein kinase C (PKC), and crosstalk between Smad1 and deltaFosB/JunD pathways synergistically stimulate *IL-11* gene transcription [48]. IL-11 suppresses the expression of the mechanical-sensitive gene *SOST* (encoding sclerostin), activates the Wnt signaling pathway, and enhances mechanical stress induced bone formation. The response of bone formation to mechanical loading is significantly reduced in global and osteoblast/osteocyte-specific IL-11^-/- ^mice [41]. The expression of the Wnt inhibitor sclerostin was increased in IL-11^-/-^ mice. Sclerostin expression was upregulated by mechanical unloading in WT mice. However, sclerostin levels remained high at baseline and under unloading and reloading conditions in IL-11^-/-^ mice. Bone formation is blunted by sustained high expression of Wnt inhibitors in IL-11^-/-^ mice under mechanical loading (32). This implies that IL-11 is a mechano-sensitive cytokine, regulating bone formation in response to mechanical loading (Fig. 2).

PTH is another stimulator of osteoblast differentiation and bone formation. PTH upregulates the expression of gp130 cytokines in osteoblasts, including IL-6, IL-11, OSMR and CRLF1 [49]. It activates the downstream signaling cascade to upregulate IL-11 expression in a time- and dose-dependent manner by stimulating osteoblast differentiation and bone anabolism in part. The effect of PTH on *IL-11* transcription is mediated by FosB/deltaFosB expression and Smad1 phosphorylation in response to PKC activation [50]. In addition, IL-11 influences endochondral bone formation during bone fracture healing. Together with bone morphogenetic protein-2, IL-11 induced osteoblast differentiation in a rabbit model of bone healing [51]. Glucocorticoid (GC) therapy is an established cause to osteoporosis [52]. It is worthwhile to note that IL-11 also suppresses osteoblast differentiation and apoptosis induced by GCs. GC downregulates IL-11 mRNA expression in osteoblasts *in vitro*, while IL-11 attenuates the inhibitory effect of GC on osteoblast differentiation [52]. However, the effect of IL-11 on ameliorating bone loss in an animal model of glucocorticoid-induced bone loss requires further investigation. Those results indicate that IL-11/IL-11R signaling is essential for osteoblast differentiation and osteogenesis.

Osteocytes have a lifespan of decades in the bone matrix, and they represent over 90% of the bone cells [53]. Their main role is to maintain bone strength. They also interact signalings with cells on the bone surface [54] and modify the local environment [55, 56]. In addition, osteocytes are sensors in response to mechanical loading, and regulate bone formation and bone mass. IL-11 expression was upregulated by mechanical stress *in vitro*. IL-11 suppressed the expression of sclerostin, an osteoblast differentiation inhibitor produced by osteocytes [43]. Thus, IL-11 promotes osteogenesis.

In osteogenesis process, mature osteoblasts produce and deposit an organic bone matrix called osteoid. The bone matrix mainly contains collagen type I. IL-11 is involved in the regulation of bone mineralization. In the human osteoblast cell line hFOB/ER, the regulation of IL-11 expression was more pronounced at the late mineralization stage of differentiation than at the earlier stages [57]. *Ex vivo* study using bone marrow stromal cells obtained from IL-11 Tg mice showed an enhanced expression of osteoblastic markers and mineralization compared to those of wild-type littermates [42]. Moreover, Monnouchi et al. demonstrated that rhIL-11 significantly increased the proportion of mineralized nodules as revealed by positive Alizarin Red staining. This implies that IL-11 promotes bone mineralization. IL-11 stimulates osteoblast differentiation through the JAK/STAT signaling pathway, leading to increased alkaline phosphatase activity and bone mineralization [58].

## IL-11 in osteoclast and bone resorption

Osteoclasts originate from hematopoietic progenitor cells and fuse into multinuclear cells during maturation. They migrate to the bone surface and undergo active bone resorption. They attach to the bone surface and form a sealing zone that regulates resorption through fission and fusion [59, 60]. Bone resorption occurs when acids and proteolytic enzymes are released to remove minerals, thereby degrading the bone matrix. The NF-kappa B (RANK)/RANK ligand signaling pathway mediates osteoclast formation and maturation. The pre-mature osteoblasts produce RANKL, which combines with RANK expressed in osteoclasts and stimulates osteoclast differentiation [61, 62].

IL-11 is secreted by osteoclasts. In an *in vitro* study, stimulating primary cultured osteoblasts with exogenous IL-11 resulted in the upregulation of RANKL expression, indicating that IL-11 is involved in osteoclastogenesis [63]. IL-11 stimulates osteoclast formation by upregulating RANKL generation in osteoblast lineage [64]. However, a recent study demonstrated that IL-11 stimulates osteoclastogenesis via a RANKL-independent mechanism [65]. IL-11 activates osteoclastogenesis in breast cancer bone metastasis-associated osteolysis through the JAK1/STAT3/c-Myc pathway [66]. IL-11 also prolongs the survival of osteoclast progenitors [67] and inhibits osteoclast maturation [38].

Although previous reports have demonstrated that IL-11 enhances osteoclastogenesis, systemic overexpression of IL-11 in Tg mice resulted in increased bone mass, specifically due to enhanced bone formation *in vivo* [42]. The IL-11R and gp130 receptors are highly expressed in mature osteoclasts; thus, mature osteoclasts respond to IL-11 [40, 68]. IL-11R-deficient mice exhibit reduced osteoclast counts, leading to decreased bone resorption [38]. Furthermore, osteoclastogenesis derived from the bone marrow of IL-11R-deficient mice shows a weaker response to exogenous RANKL stimulation than WT mice, indicating that osteoclastogenesis derived from hematopoietic lineage retards the deficiency of IL-11 signaling [38]. However, IL-11 deficient mice exhibit a slightly reduced tendency of osteoclast cell number, *RANKL* and *CathepsinK* mRNA expression in bone, but show no significant difference from the WT littermates [41]. In the IL-11 overexpressed transgenic mice, bone mass unexpectedly increased without changes in osteoclastic bone resorption [42]. Interestingly, osteoclast-specific deletion of gp130 in *Ctsk-gp130fl/fl* mice showed no differences in osteoclast number or bone resorption activity, but showed a reduction in trabecular bone and cortical growth [69]. The mechanism of gp130-mediated signaling in osteoclasts may be associated with the coupling regulation of bone modeling linking osteoblast-induced bone formation [69]. It suggests that gp130 plays a physiological role in the bone resorptive process not only through osteoclast-induced bone resorption, but also by promoting the release of coupling factors such as IL-6. IL-11 affects osteoclast formation both directly, and indirectly depending on the osteoblastic cells, just as the same family member of cytokine IL-6 acts on osteoclastogenesis directly and indirectly [42, 66, 70]. The effect of the IL-11/IL-11R axis and the hemicomplex subunit of receptor gp130 on osteoclast-associated bone resorption requires further investigation.

## IL-11 in chondrocyte

IL-11 and IL-11R are also expressed in chondrocytes, growth plates, and cartilage [71]. IL-11 stimulates cartilage damage by inducing aggrecanase activity. It causes cartilage damage encountered in pathogenesis of rheumatoid and osteoarthritis [72]. IL-11R null mice exhibit shorter length of bones, indicating that IL-11R signaling stimulates longitudinal growth in the growth plate [38]. In an animal model of rheumatoid arthritis, transduced fibroblasts were injected into the knee joints of the mice. Those animals treated with IL-11-transfected cells show reduced cartilage damage. In addition, transduction of IL-11 inhibited apoptosis in chondrocytes [73].

## IL-11 in cancer bone metastasis

IL-11 participates in the osteolytic cycle and interacts between cancer and bone cells. IL-11 is considered as osteolytic factor expressed in human breast cancer cells. Breast cancer cells with high IL-11 expression also show higher occurrences of bone metastasis [7, 66, 74]. Osteolytic bone injury process contains bone marrow homing and exosmosis, pericellular proteolysis and invasion, angiogenesis, osteoclast production, growth factor regulation, and extracellular matrix changes [75]. In a mice model of cancer-induced bone metastasis, overexpression of IL-11 in breast cancer cell lines increased tumor burden and osteolytic lesions [75]. Osteoclasts are direct mediators of bone resorption in osteolytic bone metastasis. IL-11 is a potent inducer of osteoclast formation. Notably, breast cancer cell lines target osteoblasts to stimulate IL-11 production from osteoblast, further increasing the concentration of IL-11 in the bone microenvironment [67]. It also stimulates the development and survival of osteoclast progenitor cells [67]. IL-11 may not be involved in homing of the disseminated cancer cells to bone [76]. In the bone-specific metastatic breast cancer cell line MDA-MB-231, ectopic IL-11 expression interacts with overexpression of the chemokine receptor CXC motif chemokine receptor type 4 (CXCR4) to drive osteolytic metastasis [75]. The combined overexpression of IL-11 and osteopontin (OPN, a secretory protein that stimulates the adhesion of osteoclasts to the bone matrix), significantly increases the incidence of bone metastasis [75].

Cyclooxygenase-2 (COX-2)-mediated production of IL-11 in poorly metastatic (MCF-7) and highly metastatic (MDA-MB231) breast cancer cell lines are necessary for osteolytic bone metastases to occur from breast cancer [76]. The transforming growth factor-β (TGF-β) signaling pathway also stimulates IL-11 production in combination with the COX-2 pathway [75]. TGF-β1 induces the secretion of IL-11 by activating p38-MAPK, which then enhances the transcription factor AP-1 and its binding to the promoter of *IL-11 *[74, 77]. Overexpression of *IL-11* induces osteolytic and angiogenic factors, and further promotes the upregulation of TGF-β. TGF-β rapidly induces the binding of Smad2/3 and Smad4 to the relevant regions of IL-11 and connective tissue growth factor via the canonical TGF-β /Smad pathway in metastatic cells and then participates in the bone metastasis cycle [75]. Bone matrix-induced TGF-β enhances the expression of IL-11 and other osteoclast differentiation factors in breast cancer cells, thereby further increasing the rate of bone loss [20].

Patients with breast cancer-related bone metastasis exhibit increased serum levels and mRNA expression of IL-11, suggesting that IL-11 is involved in bone metastasis via STAT3 phosphorylation [74]. IL-11 activates STAT3 induced c-Myc expression and further increases the expression of c-Fos and the nuclear factor of activated T cells 1 (NFATc1), the major regulator of osteoclastogenesis [66]. Coupled with the RANKL/RANK/OPG system, IL-11 promotes osteoclastogenesis indirectly via the stimulation of osteoblast-derived RANKL [67]. Thus, breast cancer cells produce IL-11, which in turn stimulates RANKL production in the bone microenvironment. IL-11 is essential for promoting osteolysis in breast cancer bone metastasis via RANKL-independent osteoclastogenesis by activating the JAK1/STAT3 signaling pathway [66]. Moreover, a monoclonal antibody against IL-11 blocked the osteoclastogenic effect. RANKL-dependent and IL-11 mediated osteoclastogenesis require STAT3 induced c-Myc expression. Thus, IL-11 is a possible target for the therapeutic strategy of bone metastasis.

## IL-11 in adipocyte and bone marrow

Osteoblasts originate from the pluripotent stem lineage, which also has the potential for differentiation into chondrocytes, adipocytes, and fibroblasts [78]. The Wnt signaling pathway promotes osteoblastogenesis and suppresses adipogenesis, thereby shifting the fate of MSC. Recent studies have provided insight into the role of cytokine IL-11 in adipogenesis.

**Figure 3.**
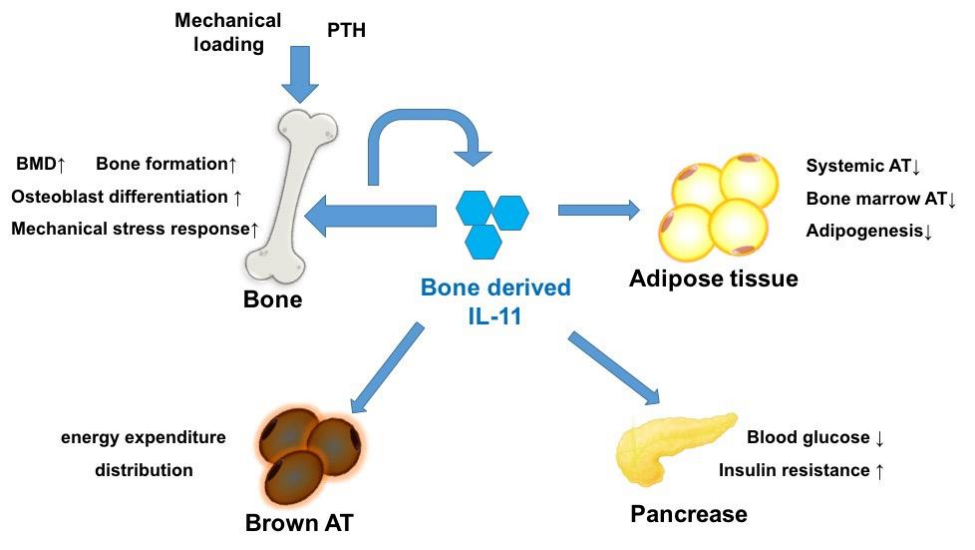
**Bone-derived IL-11 acts as an osteokine**. The osteokine IL-11 acts as bone-derived hormones, promotes bone formation and osteoblast differentiation, and increases bone mineral density (BMD) and bone enhancement response to mechanical stress. On other hand, IL-11 suppresses adipogenesis and decreases systemic and bone marrow adipose tissue (BMAT). IL-11 also improves insulin resistance and glucose homeostasis and regulates energy expenditure. The bone-derived cytokine IL-11 acts as an osteokine, linking the crosstalk among bones and other organs.

IL-11 was identified as an adipogenesis inhibitor factor early since 1990s [79-82]. In *in vitro* study, it significantly inhibited lipoprotein lipase activity and adipogenesis in 3T3-L1 cells. This suppression is controlled by tyrosine phosphorylation during the initiation of IL-11R-mediated transmembrane signaling [81]. Yang et al. demonstrated that IL-11 induces adipose-derived stem cell proliferation, migration, and anti-apoptotic effects via STAT3 signaling pathway [83]. However, research in this field has been dumped for decades. In a recent study, Dong et al. demonstrated that IL-11 suppressed adipocyte differentiation and adipogenesis via the Wnt signaling pathway. In the IL-11 systemic deletion (IL-11-/-) mice model, the systemic adiposity significantly increased (with increased adipocyte size and cell number), but with reduced bone mass and formation. In *ex vivo* study, bone marrow stromal cell derived from IL-11^-/-^ mice showed greater potential for adipocyte differentiation but not for osteoblast differentiation and osteogenesis. This phenomenon was reversed by the administration of exogenous IL-11. The expression of Wnt pathway inhibitors, SOST and Dkk1/2, was upregulated in IL-11^-/-^ mice, resulting in the suppression of Wnt signaling in adipose and bone tissues of IL-11 deletion mice. Those findings indicate that IL-11 was involved in shifting the fate of osteoblast/adipocyte differentiation cells from MSC *via* Wnt signaling pathway [41]. In addition, IL-11^-/-^ mice exhibited glucose intolerance, insulin resistance, fatty liver, and inflammatory infiltration in adipose tissue. Therefore, IL-11 influences systemic metabolism and is involved in the crosstalk between bone and other organs (Fig. 3).

## IL-11, the cytokine acts as an osteokine

The bone is considered an endocrine organ that participates in homeostasis. Bone-derived hormones, also referred as “osteokines,” link bone to adipose tissue, kidney, muscle, central sympathetic nerous system, immune system, pancreas, glucose metabolism and insulin synthesis, calcium-phosphonate homeostasis, energy metabolism, and reproduction [84].

For instance, osteocalcin is secreted by osteoblasts, and it regulates bone mineralization. Osteocalcin is an osteoblast-derived endocrine hormone involved in the regulation of multiple target organs such as pancreas, liver, muscle, testes, and nervous system [85]. Osteocalcin stimulates β-cell proliferation and insulin synthesis and increases insulin sensitivity in the liver and adipose tissues. Furthermore, osteocalcin promotes male fertility by increasing testosterone synthesis [86]. Osteocyte-derived FGF23 interacts with Klotho to influence urinary phosphate excretion, PTH, and 1,25(OH)2D3 synthesis. Thus, bone-derived FGF23 is involved in the progression of chronic kidney disease and vascular calcification [87]. Sclerostin, encoded by *SOST*, is an osteocyte-secreted protein that suppresses the canonical Wnt/β-catenin signaling pathway. It binds to the LRP5/6 receptor and inhibits bone morphogenetic protein (BMP)-2-mediated osteoblast activity [88], and bone formation response to mechanical loading [89]. Animal studies have shown that inhibiting sclerostin (using a monoclonal antibody (Scl-Ab)) increases bone formation, mineral density, and strength [88]. The monoclonal antibody of sclerostin, “Romosozumab,” is widely used for the treatment of osteoporosis in postmenopasul women [90]. Furthermore, sclerostin promotes adipogenesis [91]. In a cross-sectional study, serum sclerostin levels in patients with type 2 diabetes mellitus (T2DM) were significantly higher than those without T2DM [92]. The axis of RANK/RANKL/OPG influences bone remodeling and homeostasis [93]. RANKL forms a homotrimer and interacts with its receptor, RANK, on osteoclasts to regulate bone resorption. The RANKL/RANK/OPG axis influences bone/skeletal homeostasis, glucose homeostasis and insulin resistance. Skeletal muscles also express RANK, RANKL, and osteoprotegerin (OPG). RANKL overexpression leads to muscle atrophy. Moreover, the RANKL/OPG ratio induces cardiac hypertrophy, heart failure, and vascular calcification [94].

In a recent study, Dong et al. demonstrated that systemic IL-11 deletion mice exhibited reduced bone mass and bone formation, resulting from the suppression of the Wnt signaling pathway (41). Systemic IL-11 deletion also results in increased visceral, subcutaneous, and bone marrow adiposity. Interestingly, the conditional knockout of IL-11 in bone also results in reduced bone mass with decreased osteoblastogenesis and bone formation, similar to conventional IL-11^-/-^ mice. However, adipocyte-specific IL-11 deletion mice showed no difference in cortical or cancellous BMD compared to control mice. Increased adiposity, impaired glucose metabolism, and insulin sensitivity were observed in osteoblast/osteocyte-specific IL-11 deletion only [41]. The increased adipose tissue mass and decreased bone mass caused by the systemic deletion of IL-11 was due to a reduced osteoblast/osteocyte-derived IL-11. Those results suggest that IL-11, the cytokine derived from bone, participates as an “osteokine” in regulating bone, adipose tissue, and glucose metabolism (Fig. 3).

Osteokines are derived from bone, and they regulate bone metabolism. Furthermore, they act as hormones that link bones to other organs. IL-11 is emerging as a novel target in the treatment of bone diseases such as osteoporosis and is also involved in weight loss and glucose intolerance.

## IL-11 in bone-related human diseases

IL-11 is involved in bone-related human diseases. Tachmazidou et al. explored novel candidate genes and therapeutic targets for osteoarthritis through a genome-wide analysis of the UK BioBank database [95]. Genome-wide association analysis was performed to analyze the genetic enrichment of single-gene forms of bone diseases, as well as pathways underlying collagen formation and extracellular matrix organization. The results showed that IL-11 is associated with an increased risk of osteoarthritis and disease progression, and its expression is upregulated in osteoarthritic tissue [95]. In humans, single nucleotide polymorphisms of IL-11 (rs4252548) and IL-11R (rs11575580) lead to decreased adult height [96-98], demonstrating the role of the IL-11/IL-11R axis in skeletal development. Genetic variants in *IL-11* with mutation p.R112H are associated with osteoarthritis and a reduction in adult height [99].

Mutations of *IL-11RA* result in hereditary disorders associated with craniosynostosis, dental abnormalities, and digital malformations [100-102]. Craniosynostosis is characterized by premature synostosis of skull bone plates, with limited space available for brain tissue growth, resulting in facial and skull malformations as well as intellectual disability [103]. Generally, it occurs because of missense mutations in the extracellular domains of IL-11Rα [104-106], and these mutations are located in regions distant from the putative cytokine or receptor binding sites. Mutations in *IL-11R* impair the processing and surface expression of its receptor. Genetic analysis reveals that patients with craniosynthesis carrying homozygous high-frequency pathogenic mutations of c.662C>G (p.Pro221Arg), c.734C>G (p.Ser245Cys), c.886C>T (p.Arg296Trp) in *IL11RA* on chromosome 9p13.3 [107]. Most variants are located in the IL11Rα extracellular domains and cluster in the second Fibronectin III domain, adjacent to the C-terminal transmembrane domain. Molecular dynamics of the structure indicate that mutations in *IL-11R* destabilize the receptor and disturb the cytokine-binding region [11]. The loss-of-function of Arg296Trp mutation impairs the activation of downstream STAT3-mediated intracellular signal transduction by IL11Rα [102, 104]. Interestingly, a patient carrying a homozygous mutation in *IL6ST* (encoding gp130, p.N404Y) exhibited craniosynostosis [103]. However, no related gp130 mutant phenotypes have been identified in animal models.

In an animal study, IL-11R^-/- ^mice showed reduced long-bone length, leading to reduced body length [108]. Interestingly, 40-50% of IL-11RA^-/-^ mice exhibited craniosynostosis-like phenotypes and snout deformities, whereas no snout deformities were observed in IL-11^-/-^mice [100, 104]. In IL-11KO mice, no craniosynostosis were observed, and the BMD of the calvaria did not differ between the IL-11KO and WT mice [41].

## Conclusion

In this review, we discuss the mechanisms of IL-11 in bone metabolism and homeostasis using current knowledge from human diseases. We highlight that IL-11 acts as a bone-derived hormone, or termed as “osteokine”, and is involved in the systemic regulation linking the crosstalk between bone and adiposity. We also put insight that IL-11 promotes osteogenesis, inhibits adipogenesis, and shifts the cell fate from stromal cells. Thus, the therapeutic potential of IL-11 in osteoporosis and metabolic syndrome needs further verification.
